# Sensing Exposure Time to Oxygen by Applying a Percolation-Induced Principle

**DOI:** 10.3390/s20164465

**Published:** 2020-08-10

**Authors:** Noa Afik, Omri Yadgar, Anastasiya Volison-Klimentiev, Sivan Peretz-Damari, Avia Ohayon-Lavi, Amr Alatawna, Gal Yosefi, Ronit Bitton, Naomi Fuchs, Oren Regev

**Affiliations:** 1 Department of Chemical Engineering, Ben-Gurion University of the Negev, Beer-Sheva 84105, Israel; yadgaro@post.bgu.ac.il (O.Y.); volison@post.bgu.ac.il (A.V.-K.); sivanpe@post.bgu.ac.il (S.P.-D.); aviaoh@post.bgu.ac.il (A.O.-L.); amra@post.bgu.ac.il (A.A.); yosefig@post.bgu.ac.il (G.Y.); rbitton@exchange.bgu.ac.il (R.B.); 2 Department of Chemistry, Ben-Gurion University of the Negev, Beer-Sheva 84105, Israel; 3 The Ilse Katz Institute for Meso and Nanoscale Science and Technology, Ben-Gurion University of the Negev, Beer-Sheva 84105, Israel; 4 Department of Biotechnology Engineering, Ben-Gurion University of the Negev, Beer-Sheva 84105, Israel; naomifu@post.bgu.ac.il

**Keywords:** oxygen sensor, time sensor, clay, linseed oil, hybrid composite, irreversible sensor

## Abstract

The determination of food freshness along manufacturer-to-consumer transportation lines is a challenging problem that calls for cheap, simple, reliable, and nontoxic sensors inside food packaging. We present a novel approach for oxygen sensing in which the exposure time to oxygen—rather than the oxygen concentration per se—is monitored. We developed a nontoxic hybrid composite-based sensor consisting of graphite powder (conductive filler), clay (viscosity control filler) and linseed oil (the matrix). Upon exposure to oxygen, the insulating linseed oil is oxidized, leading to polymerization and shrinkage of the matrix and hence to an increase in the concentration of the electrically conductive graphite powder up to percolation, which serves as an indicator of food spoilage. In the developed sensor, the exposure time to oxygen (days to weeks) is obtained by measuring the electrical conductivity though the sensor. The sensor functionality could be tuned by changing the oil viscosity, the aspect ratio of the conductive filler, and/or the concentration of the clay, thereby adapting the sensor to monitoring the quality of food products with different sensitivities to oxygen exposure time (e.g., fish vs grain).

## 1. Introduction

Spoiled food is the cause of many health problems (e.g., Salmonella, Campylobacter) resulting in significant mortality [1,2,3,4,5]. Therefore, food safety along supply lines has become a major public health priority [6,7,8,9,10,11,12,13,14], with shelf life expiry being one of the main reasons for food wastage [6,10]. Lengthy manufacturer-to-consumer transportation lines increase the probability of food spoilage [11,12]. It is, therefore, essential that food manufacturers and suppliers should have at their disposal reliable real-time means for monitoring the freshness of food products [10]. One of the main factors contributing to food spoilage is the exposure to oxygen, resulting in growth of aerobic microorganisms (e.g., yeasts, molds and bacteria such as Aeromonas hydrophila, Yersinia enterocolitica) and quality degradation (change in the color, flavors and nutritional losses) [9,10,13,15,16,17,18,19]. Therefore, suitable parameters for in situ monitoring the freshness of packaged food would be exposure time to oxygen and its concentration.

### 1.1. Oxygen Sensors in the Food Industry

A widely used means of extending the shelf life of products is modified atmosphere food packaging (MAP) in which the air in the package is replaced by a gas other than air (e.g., nitrogen or carbon dioxide) [8,16,18,20]. Carbon dioxide, although released in ripening processes, is used as a replacement gas since it inhibits microbial growth (in e.g., fresh meat, fish, cheese) [18,19]. Monitoring the freshness of the food in the package is crucial and has been investigated extensively [13,17,21,22,23,24,25,26,27,28,29,30,31]. In some approaches, food spoilage is indicated by released volatiles [28,29,30,31,32] (e.g., NH_3_ for meat [30], CO_2_ for grain [31] or C_2_H_4_ for fruits [32]). While sensing volatiles is rather specific for a given food, sensing oxygen is a more general approach [13,17,21,22,23,24,25,26,27].

To date, there are two conventional sensing approaches for monitoring oxygen concentration—luminescence-based and colorimetric (based on color-change) [25,26,27]. Luminescence sensing is based on the irreversible quenching by oxygen molecules of probe molecules trapped in the polymer coating of food products [9,10]. One such system, OxySense^TM^ [33], offers a noninvasive, fluorescence-lifetime-based measurement of the oxygen concentration in packages—both in the headspace (gases) and in liquids [33,34]. This approach is relatively inexpensive and nontoxic but requires the use of an expensive analytic instrument (rather than the human eye) to determine the oxygen concentration [9]. Colorimetric methods use a simple reversible redox reaction to measure the oxygen concentration by the change in the color of the sensor [18]. However, these sensors may require storage under special conditions [10,35] and tend to be humidity sensitive and relatively expensive (e.g., Ageless Eye^TM^ [35]). The above drawbacks call for a simple and cheap approach for oxygen sensing via an irreversible reaction.

### 1.2. Bio-Based Sensors

Recently, bio-based sensors have been extensively studied due to their safety (nontoxic), economic and environmental benefits [24,36,37,38,39,40,41]. Currently, most bio-based sensors for food packages are colorimetric and indicate pH change [37,41] or dielectric constant [24].

Linseed oil is an environmentally friendly material [24,42,43,44] that is well known for its drying and hardening properties [45,46,47,48]. It consists mainly of a mixture of triglycerides of the C_18_ polyunsaturated fatty acids, linolenic (C18:3), linoleic (C18:2) and oleic (C18:1) acids [48]. Upon exposure to air, the unsaturated bonds in the acids are oxidized and cross-linked irreversibly to form a polymeric network [48]. The rate of oxidation depends on environmental conditions, such as oxygen concentration, humidity, temperature (corresponding to Arrhenius law [48,49,50]) and exposure time [24,45,48,51,52,53], and also on the degree of saturation of the oil [47,48]. It has been found that using a fatty acid in which only one unsaturated bond is oxidized will significantly decelerate (by at least factor of two) the autoxidation kinetics [45,54] compared to the initial, unsaturated state. In this respect, the oxidation rate of linseed oil is the highest among other drying oils (e.g., walnut and poppy seed oils) due to its high concentration (48–60 wt%) of the unsaturated linolenic acid (C18:3) [45,47,54]. By virtue of its industrial applicability, the properties of linseed oil – both per se and in its oxidized (polymerized) form – have been thoroughly investigated. It is known, for example, that the oxidation rate of linseed oil can be accelerated by raising the temperature to 80 ℃, without changing the cross-linking mechanism [30], whereas the humidity has a negligible effect on the oxidation rate [24,51]. The oxidation rate of the oil can be accelerated (by addition of metal-based catalysts [46]), or decelerated (by partially oxidizing the oil before use) yielding, e.g., thickened linseed oil [55,56,57] with higher viscosity [48]. A different approach for decelerating the oxidation rate involves loading the oil with a filler that acts as a gas barrier [58,59], hence reducing the oxygen permeability [60,61,62] and consequently the oxidation rate [24,45,63]. The above-described principles have indeed been applied in a linseed-oil-based oxygen sensor, which relied on tracking the decrease in the dielectric constant of the oil upon exposure to oxygen [24]. However, this approach requires the development of a disposable radio-frequency identification interface to analyze the readout of the sensor. Moreover, its detection is limited only to a few hours after exposure to the gas.

### 1.3. Composite-Based Oxygen Sensors

The sensor proposed here rests on the concept of a composite material—a mixture of two or more materials with significantly different physical or chemical properties in one structural unit that comprises a continuous part (matrix) loaded with filler(s) [64,65,66,67,68]. The choice of the dispersed fillers is guided by their intrinsic and structural (e.g., size and aspect ratio—the length of the filler divided by its width) properties that are expected to enhance the properties (e.g., mechanical, electrical conductivity and permeability) of the composite [67,69,70,71,72]. Polymer composite materials have previously been employed as gas sensors in which the permeating gas causes the polymer to swell, thereby decreasing its electrical conductivity (EC) [71,73,74,75,76]. Those studies focused largely on reversible sensors that are not suitable or sufficiently reliable for food package sensing [9,10,73,74,75,77]: when a reversible sensor in a food package is exposed to oxygen, microbial growth converts some of the oxygen to carbon dioxide, causing the sensor to provide an erroneous (lower concentration) signal that does not reflect the freshness of the food [9].

Our suggested EC-based oxygen sensor is prepared by adding a graphite powder as a conductive filler to a linseed oil-clay composite. This new composite sensing material is based on a two-pronged principle: (i) polymerization (and shrinking) upon exposure to oxygen, and (ii) percolation of the conductive filler and increase in EC due to the polymerization of the linseed oil matrix. Unlike the classic approaches that are based on sensing the *oxygen concentration*, our composite-based sensor is designed to alert the consumer as to the *duration of exposure to oxygen*.

While for some food products (e.g., meat, bread) exposure of several days to the oxygen in the air is harmful, for others (e.g., grain, butter) even exposure of several weeks is harmless [20,78]. We aim to design a tailor-made oxygen sensor that is tuned to set off at the food type specific spoilage time.

The proposed sensor is nontoxic, cheap and easily prepared; it induces an irreversible response; and it is tunable in that its temporal EC can be manipulated by judicious choice of the oil (e.g., refined vs thickened linseed oil), the type of graphite powder (in terms of the aspect ratio), and/or the concentration of clay.

## 2. Materials and Methods

### 2.1. Materials

The electrically conductive filler comprised either Graphite Powder (Alfa Aesar, Ward Hill, MA, USA; crystalline, 300 mesh [69,70]; designated here as GPA) or Graphite Powder BTC (BeanTown Chemical, Hudson, NH, USA; crystalline, 300 mesh; designated GPB). The clay filler, Cloisite 20A (Nanoclay, BYK, Geretsried, Germany), comprised a natural montmorillonite clay that has been hydrophobically modified with a quaternary amine connected to two long hydrophobic hydrocarbon chains (hydrogenated tallow) [60,79,80,81]. As the matrix, refined linseed oil or thickened linseed oil (Winsor & Newton, London, UK) were used. The major relevant properties of these materials are summarized in Table 1.

### 2.2. Composite Preparation

A linseed oil matrix loaded with filler(s) was poured into a 20-mL glass vial and mixed in a planetary centrifugal mixer (2000 rpm, room temperature and atmospheric air, ARE-100, Thinky, Tokyo, Japan). A zirconia ball (5 mm diameter) was added to the mixing container to enhance the shear forces during the mixing process. Mixing was performed twice (1 min each time with a 2 min break) to prevent heating of the composite due to the intense shear forces. The zirconia ball was removed after this process.

### 2.3. Characterization of the Oil and the Composite

#### 2.3.1. Gas Chromatography with Flame-Ionization Detection

The composition of the oil was determined by gas chromatography with flame-ionization detection (GC-FID, model 7890A, Agilent, Santa Clara, CA, USA). The refined linseed oil sample (40 µL) was suspended in KOH/methanol solution (0.5 N) to separate the fatty acids from the triglycerides. Fatty acid methyl esters (FAMEs) were extracted from the reaction mixture with hexane and analyzed in a gas chromatograph equipped with a flame ionization detector (model 7890A, Agilent, Santa Clara, CA, USA). FAMEs were separated in a DB-23 capillary column (60 m, 0.25 mm, 0.25 µm, Agilent, Santa Clara, CA, USA) with hydrogen as the carrier gas. The composition of the different fatty acids was calculated as the ratio of the area of a given fatty acid peak to the overall area.

#### 2.3.2. X-ray Photoelectron Spectroscopy

Elemental analysis of the neat oil was performed with an X-ray Photoelectron Spectrometer (XPS; ESCALAB 250, Thermo Fisher Scientific, Loughborough, UK), operated at ultrahigh vacuum (10^−9^ bar) and employing an Al Kα X-ray source and a monochromator. The X-ray beam diameter was 500 μm, and survey spectra were recorded with a pass energy of 150 eV. To correct for charging effects, all spectra were calibrated relative to the carbon C 1s peak positioned at 284.8 eV. The XPS results were analyzed using the AVANTGE program. The accuracy of the measurements was 0.2 atomic percent.

#### 2.3.3. Rheology

A rheometer (AR 2000, TA Instruments, New Castle, DE, USA) with a 40 mm 4° steel cone configuration was used to measure the zero-shear viscosity (at 0.1 Hz shear rate) of the neat oils and the composites at 25 ℃. The accuracy of the measurement was 5% of the measured value.

#### 2.3.4. Scanning Electron Microscopy

The fillers were imaged by a high-resolution field-emission gun Scanning Electron Microscope (SEM; JSM-7400F, JEOL, Tokyo, Japan) operated in a backscattered electron mode (AUTRATA YAG, AutraDet, Brno, Czechoslovakia). SEM samples were coated with a 5 nm Au film by spattering (EmitechK575X, Emitech Ltd., Ashford, Kent, UK).

#### 2.3.5. Electrical Conductivity

Immediately after the preparation of the composite, it was deposited on a flame-retardant printed circuit board (PC-12106A, gold-coated electrodes, Aviv PCB&Technologies, Yoqneam Moshava, Israel) using doctor blade, in an area of 0.5 × 1.0 cm^2^ delineated by polyimide tapes (90 µm thickness, 5413, 3M^TM^, St. Paul Minnesota St. Paul, MN USA) as shown in Figure S1 in the Supplementary Materials.

The temporal resistance was measured with an applied potential of 20 V by picoammeter (B2985A Keysight, Mansfield, TX, USA, sensitivity of 10^−12^ S/cm) at 27 °C, atmospheric air (21% oxygen) and relative humidity of 40%. The effect of humidity has a negligible effect on the oxidation rate [24,51] and therefore was not studied. In addition, linseed oil under oxygen-free atmosphere, e.g., N_2_(g) environment, did not react at all during 250 h indicating the significance of oxygen presence for the polymerization [52]. The oxidation rate of linseed oil agrees with Arrhenius law [48,82,83] as well as spoilage of some food products [9,78,84], therefore all the measurements were conducted under the same temperature, 27 °C, humidity and oxygen concentration.

The resistance, *R* (ohm, Ω) measurements were repeated three times with three different specimens, and the EC and the error bars (standard deviation) were calculated using the equation: EC$\;\left[{S\;\mathrm{cm}}^{-1}\right]=1/\rho =d/R\cdot S\;,$ where *ρ* [Ω cm^−1^] is the electrical resistivity, *S* (cm^2^) is a cross-sectional area perpendicular to the current direction and *d* (cm) is the sample thickness.

#### 2.3.6. Thermogravimetric Analysis

A thermogravimetric analyzer (TGA; STDA851, Mettler-Toledo, Greifensee, Swiss) was used to evaluate the drying rate. Each run was conducted in an air atmosphere (50 mL/min) at 80 °C with relative humidity of 40% and lasted for 72 h.

#### 2.3.7. X-ray Scattering

X-ray scattering (300-XL, SAXSLAB (Xenocs)’s GANESHA, Grenoble, France) was performed under vacuum at room temperature with Cu Kα radiation (generated by a Genix 3D Cu-source), an integrated monochromator, three-pinhole collimation, and a two-dimensional Pilatus 300K detector. The scattering intensity was recorded in the 1° to 25° 2θ interval. The scattering curves were corrected for counting time and sample absorption. The composite specimens were placed in stainless steel sample cells with mica entrance and exit windows. Graphite and clay powders were placed in a 1.5-mm quartz glass capillary (wall thickness of 0.01 mm). The 2D images were azimuthally averaged to produce one-dimensional profiles of intensity, I, versus 2θ, using the two-dimensional data reduction program SAXSGUI.

## 3. Results and Discussion

We developed a novel hybrid composite-based sensor consisting of a linseed oil matrix loaded with graphite powder and clay fillers. Two types of linseed oil (refined and thickened), differing in their viscosity and degree of saturation, and two graphite powder fillers (GPA and GPB) differing in their aspect ratio, were examined (Table 1). The pristine oils and fillers were first characterized by GC-FID, XPS, rheology measurements and SEM. Upon exposure to oxygen, the linseed oil undergoes oxidation, polymerization and consequent shrinkage, causing the concentration of the electrically conductive graphite powder to increase up to and beyond the EC percolation. This process, if well calibrated, can be exploited to indicate food spoilage. Therefore, temporal EC measurements of the composite-based sensor were carried out under air, and the duration of the sharp increase in the EC was expressed as a parameter that we defined as an 'indication of air-exposure time' (IAET). To determine the optimal sensor composition, we explored the effects of the viscosity of the oil, the aspect ratio of the conductive filler (graphite powder), and the loading of the thickening filler (clay) on the EC percolation. The addition of clay (as an insulating filler) produced an excluded volume phenomenon, in which the EC of the composite increased with the concentration of the clay. Our proof-of-concept study indicates that by judicious choice of the degree of saturation of the matrix, the aspect ratio of the conductive filler and the concentration of the clay, the composite-based sensor could be tuned to provide a warning regarding the spoilage of a given food product.

### 3.1. Characterization

#### 3.1.1. Matrix Oil Composition and Viscosity

Analysis of the fatty acid composition of the refined linseed oil by GC-FID (Table 2 and Figure S2 in the Supplementary Materials) indicated a high concentration of linolenic acid (C18:3-9,12,15), in line with the supplier’s specification [45]. XPS revealed only organic materials, with no traces of metal-based catalyst (Table 3 and Figure S3 in the Supplementary Materials) that could accelerate the oxidation process, and hence influencing the EC [47,48]. The thickened linseed oil, being a slightly polymerized refined oil, had a higher viscosity (by a few orders of magnitude) than the refined linseed oil (Table 1), reflecting the difference in the degree of saturation of the two types of oil [48,55].

#### 3.1.2. SEM Imaging of the Fillers

**Figure 1:**
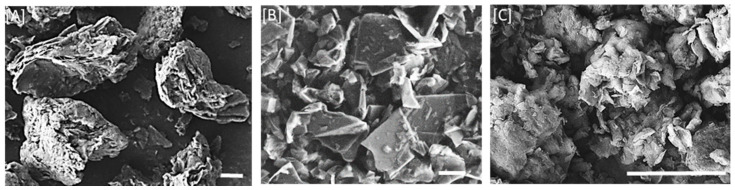
SEM micrographs of (**A**) graphite powder Alfa Aesar (GPA) × 430, (**B**) graphite BTC (GPB) × 1000, and (**C**) clay × 5000; scale bar = 10 µm.

Scanning electron microscopy (SEM) of the Graphite Powders reflected the differences in the aspect ratios (Table 1) between the isotropic, sphere-like Alfa Aesar graphite (GPA, Figure 1A) and the plate-like BTC graphite (GPB, Figure 1B), which had a much higher aspect ratio (Table 1). SEM of Cloisite 20 A revealed stacked thin layers of clay (Figure 1C).

### 3.2. The 'Shrink-Percolate' Approach

**Figure 2:**
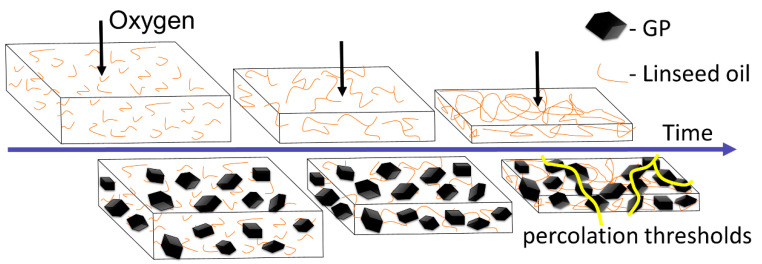
Schematic representation of the oil shrinkage mechanisms. Upper and lower panels depict shrinkage in the absence and presence of a conductive filler, respectively. The yellow lines indicate conductance paths. GP = graphite powder.

Our proposed sensing concept is based on oxidation-induced shrinkage of the linseed oil matrix and percolation of the conductive filler, i.e., graphite powder (Figure 2). The exposure of linseed oil to atmospheric air (21% oxygen) initiates its polymerization [45,48] through cross-linking of the fatty acid chains in which the weak and relatively long van der Waals bonds between the chains are converted to shorter covalent bonds, resulting in matrix shrinkage. When matrix shrinkage takes place in the presence of conductive fillers, such as graphite powder, it will result in electrical percolation induced by the increase in the concentration of the graphite powder, as was obtained here (Figure 2).

### 3.3. Effect of the Degree of Saturation of the Oil on the Electrical Conductivity

**Figure 3:**
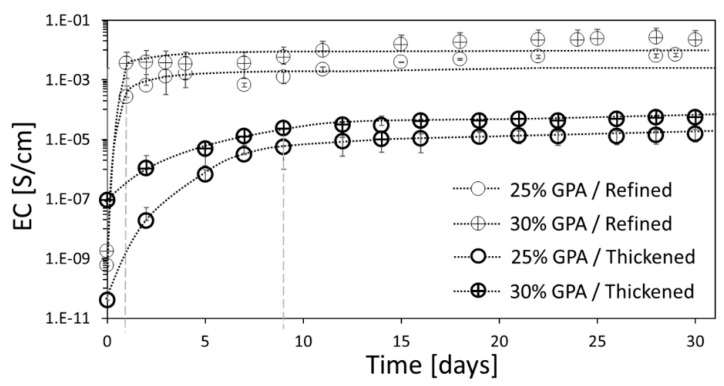
Temporal electrical conductivity (EC) of refined and thickened linseed oils loaded with different concentrations of GPA upon exposure to air at 27 °C. The dashed lines denote the indication of air-exposure time (IAET) in which the composite ended the shrink-percolate process (one or nine days for the refined and thickened linseed oils, respectively). The dotted lines are drawn as guidance for the eye. The error bars are shifted to low EC values due to the logarithmic scale.

We measured the temporal EC of air-exposed linseed oil (both refined and thickened) loaded with GPA and evaluated the time required for shrinking-induced percolation to occur (Figure 3). The initial GPA concentration was below the EC percolation threshold of 35 wt% (Figure S4 in the Supplementary Materials). A sharp increase in EC (ending after one or nine days for the refined and thickened linseed oils, respectively) was followed by a plateau (Figure 3). The duration of the sharp increase in EC (dashed lines in Figure 3) was defined as an Indication of Air-Exposure Time (IAET), after which the food is spoiled. Food spoilage can take several days or even weeks [20,78,85], and we therefore sought the means to tune the IAET value such that it could be exploited as a sensor for a variety of packaged foods.

Both oil types showed a significant increase in EC, of several orders of magnitude, which was easily detectable. The refined linseed oil systems showed higher EC values (by two to three orders of magnitude) than the thickened systems due to the higher viscosity of the latter (Table 1), which restricted the mobility of the fillers [72,86] and thus required a higher concentration of conductive filler for the EC percolation threshold to be reached. Therefore, for the same filler concentration, the viscous thickened oil had lower EC, in keeping with previous reports [65]. The thickened oil exhibited a more moderate EC increase than the refined oil due to its slower oxidation kinetics, as dictated by the lower concentration of unsaturated chains (Section 3.1.1), which reduced the probability and the reaction rate of cross-linking (shrinking) [45,54].

### 3.4. Effect of the Aspect Ratio of the Graphite Powder on the Electrical Conductivity

**Figure 4:**
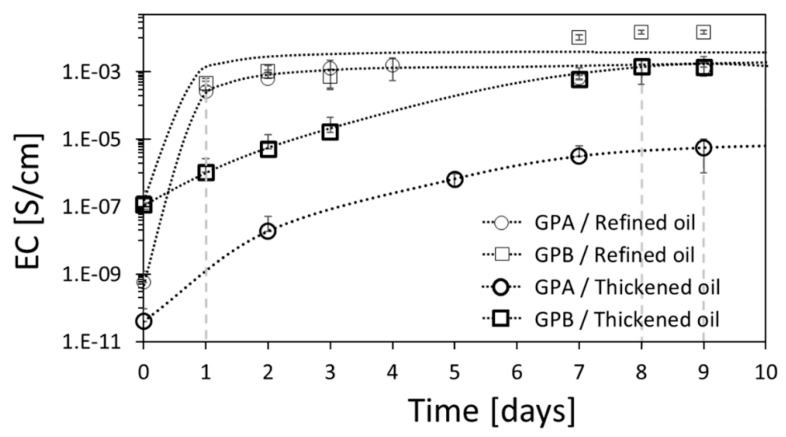
Temporal EC of refined and thickened linseed oils loaded with 25 wt% GPA or GPB upon exposure to air at 27 °C. The dashed lines denote the indication of air-exposure time (IAET) during which the composite ended the shrink-percolation process. The dotted lines are drawn as guidance for the eye.

The oxidation kinetics of graphite-powder-loaded (either with GPA or GPB) composites was characterized by a marked increase in EC over the first few days (up to the IAET), followed by a plateau at the final EC value (Figure 4). For both oil types, the IAET was only mildly affected by the type of graphite powder, giving one and eight or nine days for the refined and thickened oils, respectively. Nevertheless, the plateau EC value for the GPB–refined oil composite was highest due to the higher aspect ratio of GPB, promoting the formation of the EC conducting path [87].

### 3.5. Clay Addition to Form a Hybrid Composite System

**Figure 5:**
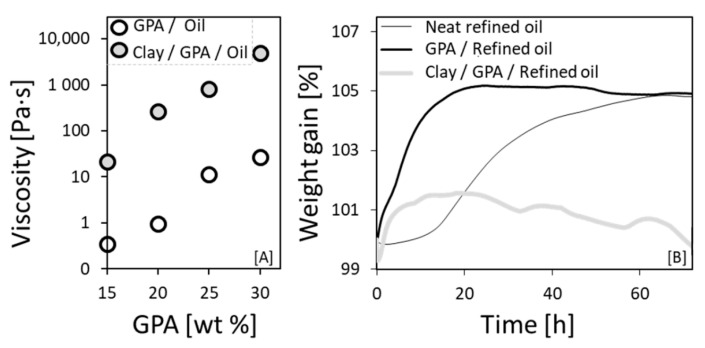
Effect of clay on the linseed oil–graphite powder composite: (**A**) Zero-shear (at 0.1 Hz shear rate) viscosity of thickened linseed oil loaded with GPA (with and without 10 wt% clay) vs GPA concentration, measured immediately after preparation. (**B**) Accelerated aging studied by thermogravimetric analysis (at 80 °C, in air) of refined linseed oil: neat, and loaded with single (30 wt% GPA) or hybrid (30 wt% GPA–25 wt% clay) fillers. The neat oil served as a reference.

To maintain the integrity of the sensor (i.e., keeping a constant film thickness and avoiding dripping) during transportation, a certain viscosity of the composite material is required. Therefore, to increase the viscosity of the composite, we added to the graphite-powder-loaded oil a second nonconductive filler material, clay (Cloisite 20A) [58,59,60]. Due to the hydrophobic nature of the modified clay (see Materials Section 2.1), it dispersed easily in the thickened linseed oil via intercalation within the galleries of the clay, and it significantly increased the viscosity of the composite (Figure 5A) [60,88]. An additional reason for the choice of clay as the thickening component was that clay acts as a gas barrier [63], reducing the rate of oxygen permeation in the composite; this property can be exploited in sensors for long shelf-life food products that require a long IAET (Section 3.3). The influence of the addition of clay to the composite system was studied by following thermograms of accelerated oxidation reactions (80 ℃, air atmosphere) in which the long aging process is shortened without changing the oxidation mechanism [46] (Figure 5B). The measured weight gain of the neat refined oil (due to oxidation) was ~5% after 65 h, in agreement with previous reports [45], while the linseed oil-GPA composite reached the same value after only 18 h, indicating a GPA-catalyzed oxidation reaction [89,90]. However, upon clay addition to the system, the oxidation rate was substantially decreased (weight gain of only ~1.5%), in keeping with the gas-barrier properties of the clay.

#### 3.5.1. Clay-Induced Excluded Volume Effect

**Figure 6:**
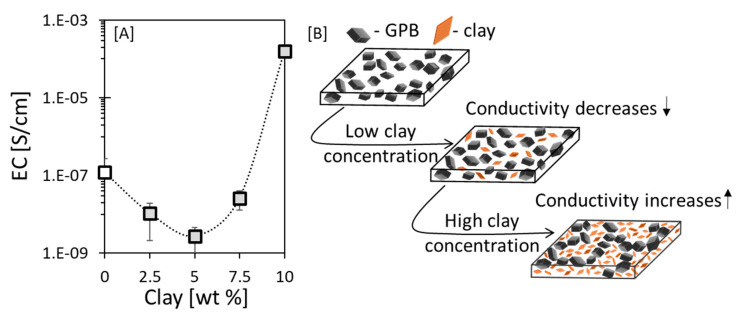
(**A**) Clay concentration-induced change in the EC of a GPB-thickened oil system measured immediately after preparation with a fixed GPB concentration of 25 wt%. The dotted lines are drawn as guidance for the eye. (**B**) Schematic representation of thickened linseed oil (white) loaded with GPB (black) and clay (orange). At low clay concentration, the EC decreased since the nonconductive clay particles impeded the formation of conductive network. At higher clay concentration the EC increases due to excluded volume effect forcing the conductive GPB fillers to percolate.

We continued to explore the EC of the proposed sensor in experiments using the *GPB-thickened linseed oil hybrid system*. Our choice of system for these experiments was based on: (i) the GPB filler (by virtue of its higher aspect ratio vs GPA) yielded composites with relatively higher EC plateau values (Figure 4), which were easily detectable, and (ii) thickened linseed oil has a higher viscosity (vs refined oil), which prevents agglomeration and stabilizes the fillers in the oil matrix [88]. The thickened oil system loaded with 25 wt% GPB exhibited a relatively low EC immediately after preparation (~10^−7^ S cm^−1^; Figure 6A). Upon addition of a low concentration of clay (<5 wt%), the EC decreased even further, since the nonconductive clay particles [59] impeded the formation of the conductive network by the conductive graphite powder (Figure 6B, middle panel). However, at a higher clay concentration (> 5 wt%), there was an increase in the EC, most probably due to the excluded volume effect by which the added volume of clay particles forced the conductive GPB fillers to percolate (Figure 6B, lower panel).

**Figure 7:**
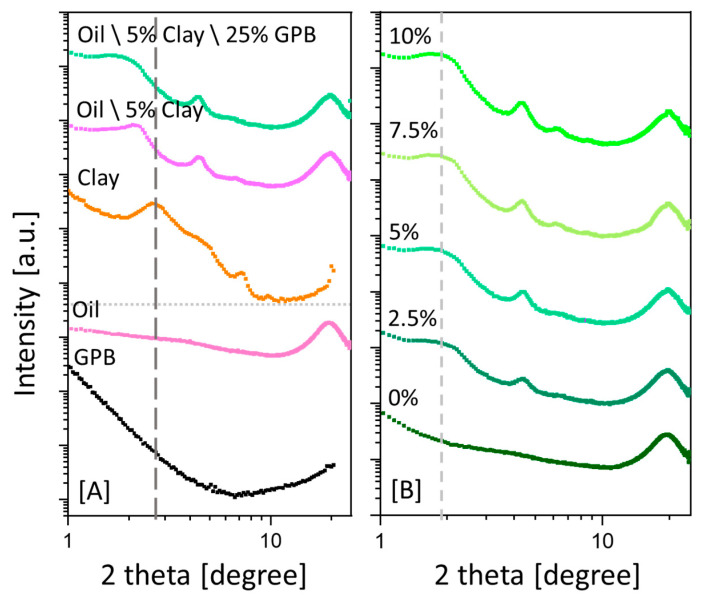
(**A**) X-ray scattering curves of thickened oil loaded with clay and GPB fillers, indicating oil intercalation between the galleries of the clay. The dashed line denotes the major clay reflection as a reference. The scattering curves of the oil and GPB alone (bottom) are presented as a reference. The major oil reflection (19.6°) refers to the hydrogen bond interactions that are not affected by the addition of the different fillers. (**B**) X-ray scattering curves showing that the concentration of the clay (at fixed 25 wt% GPB) did not affect its d-spacing: the major reflection of the swollen clay is denoted by a dashed line. All scattering curves were measured immediately after preparation.

The interaction of the oil with the clay (5 wt%) was studied by x-ray scattering; the plot shows a shift in the major clay reflection (dashed line in Figure 7A) to a lower angle (and higher d-spacing, from 3.36 to 4.13 nm) as a result of oil intercalation within the galleries of the clay (Figure 7). This resulted in clay swelling (Figure 7A), in agreement with previous studies on clay swollen with different polymers [61,62,79,91]. The addition of 25 wt% GPB to the oil–clay system broadened the basal spacing peak (indicating a wider polydispersity) but did not substantially increase the basal spacing (from 4.13 to 4.36 nm, Figure 7A). This finding implies that the interactions between the clay and the oil were not disrupted by the presence of the graphite powder and that the graphite powder did not adopt an ordered structure due to the clay-induced crowding [62,91].

In addition, the GPB-thickened oil system was loaded with different clay concentrations (Figure 7B), as was done for the EC measurement (Figure 6A). At 0 wt% clay, the scattering curve showed only the reflection of the oil (19.6°). Loading the oil–GPB dispersion with higher concentrations of clay indicated a fixed clay spacing of about 4.36 nm, irrespective of the concentration (dashed line in Figure 7B), implying that the main reason for the change in the EC (Figure 6) was the excluded volume effect and not oil–filler interactions.

#### 3.5.2. Manipulation of the IAET

**Figure 8:**
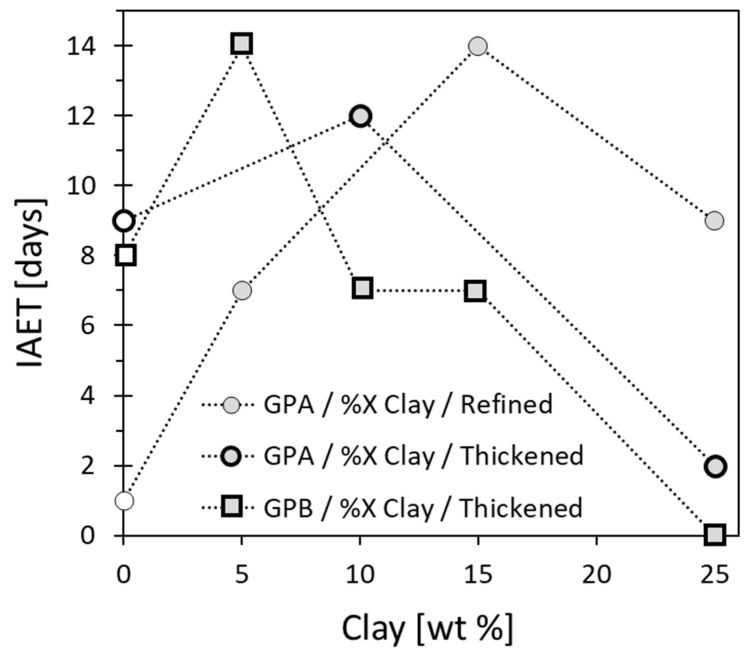
IAET as a function of clay loading at fixed graphite powder concentration of 25 wt%, for the two types of oil, refined and thickened, and for two types of graphite powder, GPA and GPB. All the IAETs are based on EC measurements (Figures S5–S7 in the Supplementary Materials) measured at 27 °C, atmospheric air (20% oxygen) and 40% RH. The dotted lines are drawn as guidance for the eye.

Since the ‘alert’ time for food spoilage (IAET, Section 3.3) could range from several days to weeks [78,85] (depending on the food type), the IAET should be adjusted accordingly to the desired food type by judicious choice of oil or graphite powder types and the concentrations of the clay and the graphite powder. Therefore, we measured the temporal EC of air-exposed linseed oil (refined or thickened) loaded with a fixed graphite powder (GPA or GPB) concentration of 25 wt% and different clay concentrations, and evaluated the IAET (Figure 8 and Figures S5–S7 in the Supplementary Materials). In all the systems, the IAET became longer with increasing clay concentrations, due to decreased oxygen permeability (Section 3.5), which served to decelerate the oxidation rate of the oil. Above a maximal clay concentration, a decrease in IAET was observed in all the studied systems (Figure 8) due to the excluded volume effect, resulting in graphite powder (both GPA and GPB) percolation (Figure 6).

In the absence of clay, the effect of the aspect ratio of the graphite powder (Table 1) on the IAET of the composite was rather mild (Figure 4 [clay] = 0, Section 3.4), but addition of clay pushed the system closer to the percolation threshold, thereby increasing its sensitivity to differences in the aspect ratio of the graphite powders. Therefore, GPB, with the higher aspect ratio, has a higher probability to form a conductive network, resulting in percolation at a lower clay concentration, i.e., a shorter IAET [87]. Indeed, the maximal IAET for the GPB-loaded thickened oil system occurred at a lower clay concentration compared to the GPA-loaded system (Figure 8). In addition, the maximal IAET value of the thickened oil–GPA system occurred at a lower clay concentration than that for the refined oil–GPA system. A possible reason for this difference was the significant increase in composite viscosity of the thickened oil (Figure 5), which decelerated the shrinkage effect. For the thickened system, the composite material became solid-like at high clay concentrations, which resulted in rather low IAET values (zero and two days for GPB and GPA, respectively). However, the viscosity of the refined oil was much lower (Table 1) and therefore the composite exhibited IAET values of nine days at the same clay concentration.

In summary, the IAET values for various compositions of the proposed sensor (Figure 8) could serve as a road map for sensor design. Shorter or longer IAET values could be chosen according to the food type, e.g., fish or coffee, respectively.

## 4. Conclusions

We have developed oxygen sensors based on linseed-oil–clay–graphite-powder composites designed to track the exposure time to oxygen rather than the oxygen concentration per se. We have shown that in the presence of oxygen the oil shrinks and percolates (i.e., a sharp increase in EC) induced by the increase in the concentration of the graphite powder. The time at which percolation occurs can be used as an indication of air exposure time (IAET), which relates to food spoilage.

The duration required to reach percolation can be tuned to match different IAETs, thus serving as a food spoilage sensor for a variety of food products. We found that changing the viscosity of the linseed oil from refined to thickened shortened the IAET while increasing the aspect ratio of the conductive filler increased the EC values (easier to detect) and reduced the IAET. Surprisingly, the concentration of the nonconductive clay affected the IAET; at low clay concentrations, the IAET was longer due to lower oxygen permeability; at high clay concentrations the nonconductive clay enhanced the EC of the composite via an excluded volume mechanism, through which the conductive fillers were forced to percolate. The summary of the combined effects of these parameters on the IAET could serve as a road map for sensor design.

## Figures and Tables

**Table 1:** Relevant properties of the materials: zero shear viscosity (at 0.1 Hz shear rate) of the matrix, mean particle size and aspect ratio (i.e., length divided by the width) of the fillers.

	Viscosity [Pa S]	Mean Particle Size [µm]	Aspect Ratio
Refined linseed oil	0.039 ± 0.002		
Thickened linseed oil	2.4 ± 0.1		
GPA [69,70]		27 ± 4	1.5 ± 0.4
GPB		10.0 ± 0.2	39 ± 6
Clay—Cloisite 20A [80,81]		6.0 *	192—2800 **

* median value; ** after sonication in toluene.

**Table 2:** Composition of fatty acids in refined linseed oil, as extracted from Figure S2.

	Palmitic C16:0	Stearic C18:0	Oleic C18:1 cis-9	Linoleic C18:2 cis-9,12	Linolenic C18:3 cis-9,12,15
**Area %**	6.1	5.0	20.5	15.6	45.1

**Table 3:** Binding energy and atomic percent of refined linseed oil, as shown by XPS (Figure S3).

Peak	Binding Energy [eV]	Atomic %
O1s	529.44	16.28
C1s	281.84	83.72

## Supplementary Materials

The following are available online at https://www.mdpi.com/1424-8220/20/16/4465/s1, The supplementary materials to this article include picture of the sensor, XPS measurements, examination of the GPA concentration for the EC percolation and temporal EC of the different compositions.

## Abbreviations

EC	Electrical Conductivity
MAP	Modified Atmosphere Packaging
GPA	Graphite Powder Alfa Aesar
GPB	Graphite Powder BTC
IAET	Indication of Air-Exposure Time
