# Supplementary material for: Sensing Exposure Time to Oxygen by Applying a Percolation-Induced Principle
Source: Sensors (Basel). 2020 Aug 10;20(16):4465. doi: 10.3390/s20164465 (PMC7471990; doi:10.3390/s20164465)
Supplement: Supplementary file 1 [file sensors-20-04465-s001.pdf]

Supplementary Materials

Sensing Exposure Time to Oxygen by Applying a Percolation-Induced Principle

Noa Afik [1,2,*], Omri Yadgar [1], Anastasiya Volison-Klimentiev [1], Sivan Peretz-Damari [1], Avia Ohayon-Lavi [1], Amr Alatawna [1], Gal Yosefi [1], Ronit Bitton[1,3], Naomi Fuchs [4], Oren Regev [1,3] *

1 Department of Chemical Engineering, Ben-Gurion University of the Negev, Beer-Sheva 84105, Israel; yadgaro@post.bgu.ac.il (O.Y.); volison@post.bgu.ac.il (A.V.-K.); sivanpe@post.bgu.ac.il (S.P.-D.); aviaoh@post.bgu.ac.il (A.O.-L.); amra@post.bgu.ac.il (A.A.); yosefig@post.bgu.ac.il (G.Y.)

2 Department of Chemistry, Ben-Gurion University of the Negev, Beer-Sheva 84105, Israel

3 The Ilse Katz Institute for Meso and Nanoscale Science and Technology, Ben-Gurion University of the Negev, Beer-Sheva 84105, Israel; rbitton@exchange.bgu.ac.il

4 Department of Biotechnology Engineering, Ben-Gurion University of the Negev, Beer-Sheva 84105, Israel; naomifu@post.bgu.ac.il

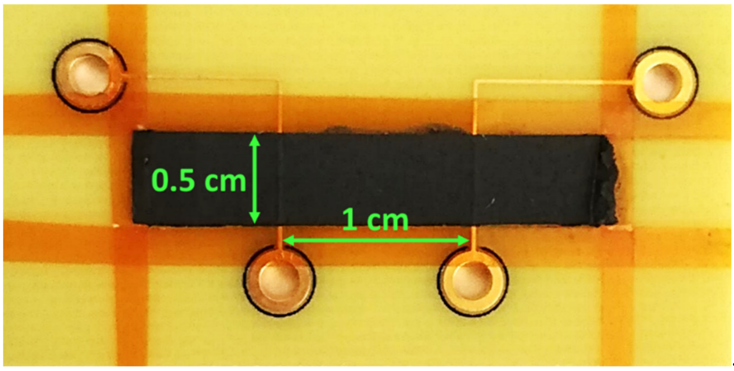

Figure S1. Picture of the sensor. A flame-retardant printed circuit board (yellow), with the smeared composite material (black), delineated by polyamide tape (orange). The parameters required for calculation of electrical conductivity are denoted in green.

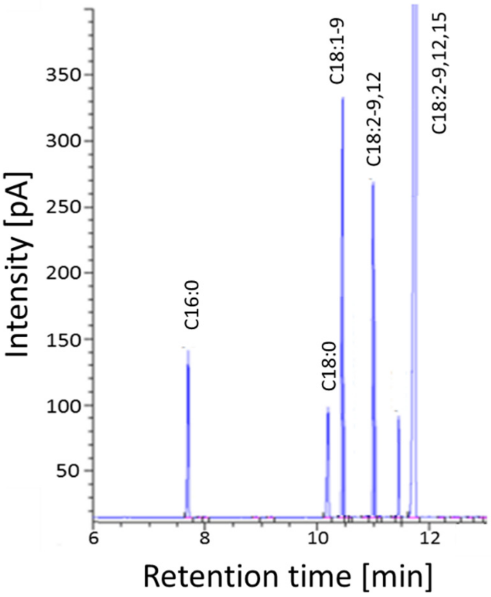

Figure S2. A GC-FID chromatogram (intensity vs retention time) of refined linseed oil.

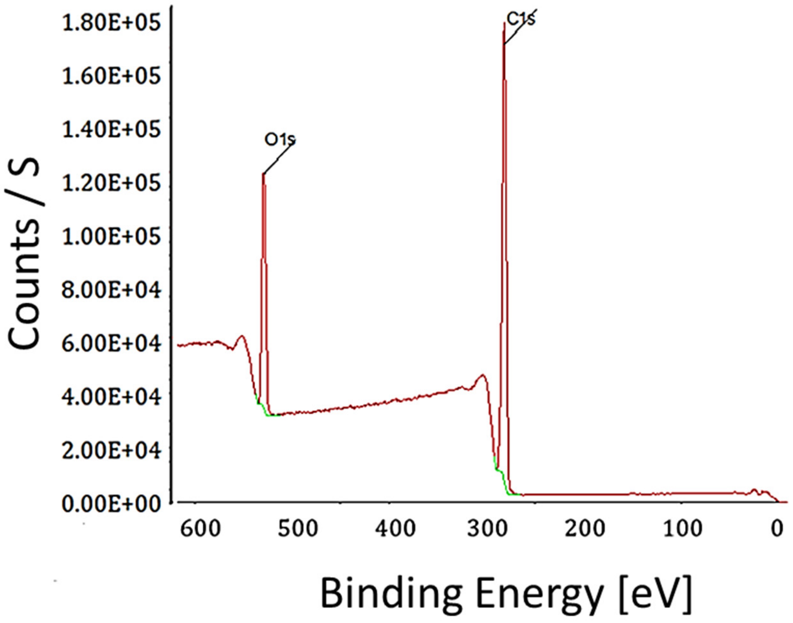

Figure S3. XPS measurement of the pristine (filler-free) refined linseed oil.

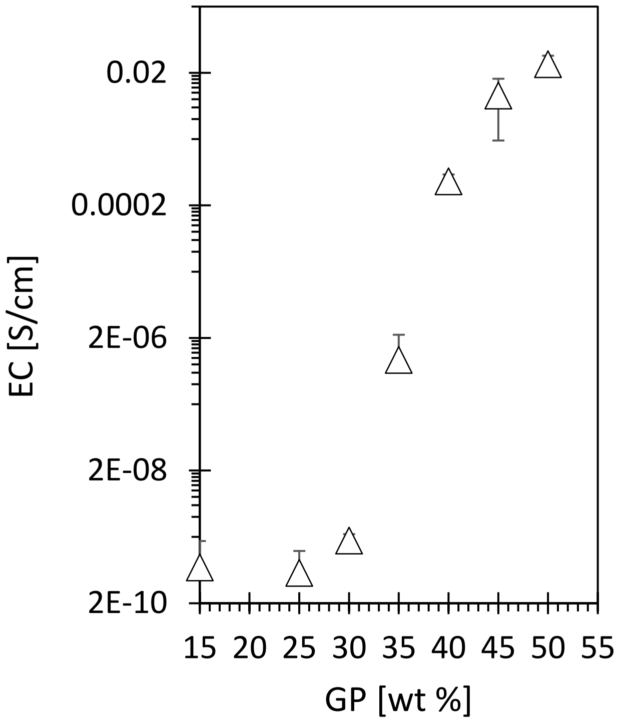

Figure S4. EC of refined linseed oil vs GPA concentration measured immediately after sample preparation (t = 0). The percolation concentration is 35 wt% GP.

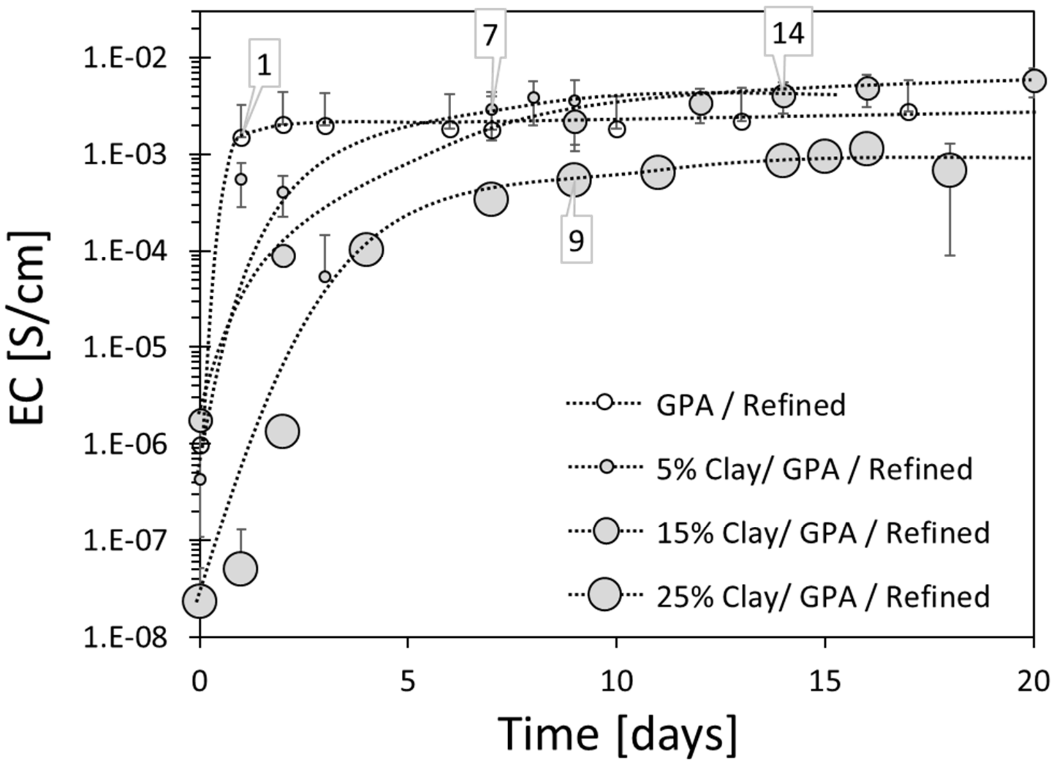

Figure S5. Temporal EC of refined linseed oil loaded with a fixed GPA concentration of 25 wt% and different concentrations of clay upon exposure to air at 27 ℃. The numbers inserted as labels on the curves are the IAETs of each hybrid system. The dotted lines are drawn as guidance for the eye.

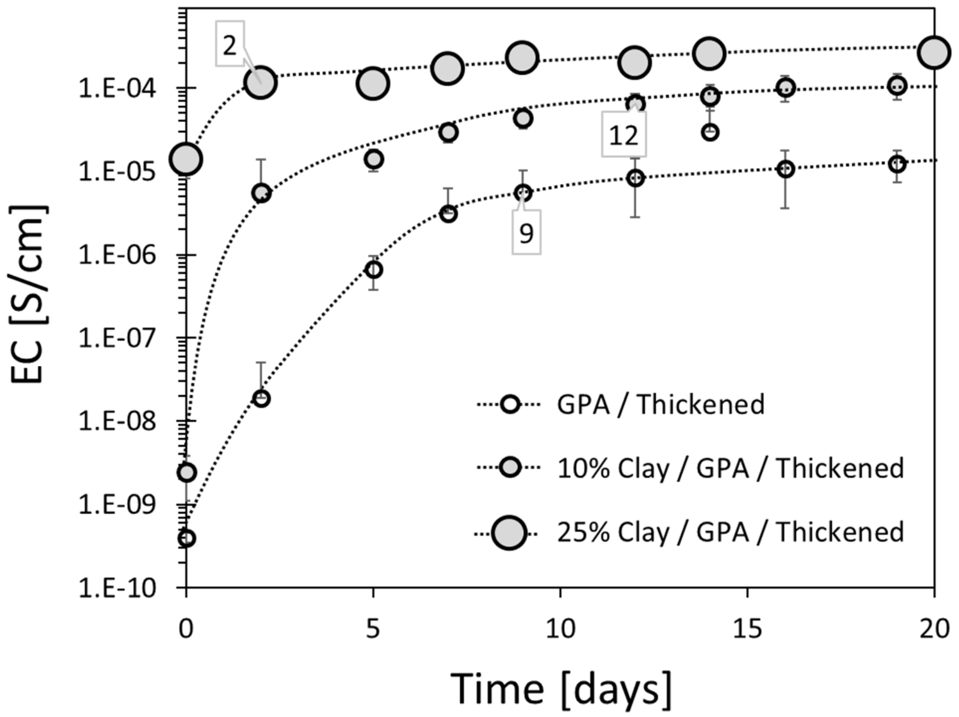

Figure S6. Temporal EC of thickened linseed oil loaded with a fixed GPA concentration of 25 wt% and different concentrations of clay upon exposure to air at 27 ℃. The numbers inserted as labels on the curves are the IAETs of each hybrid system. The dotted lines are drawn as guidance for the eye.

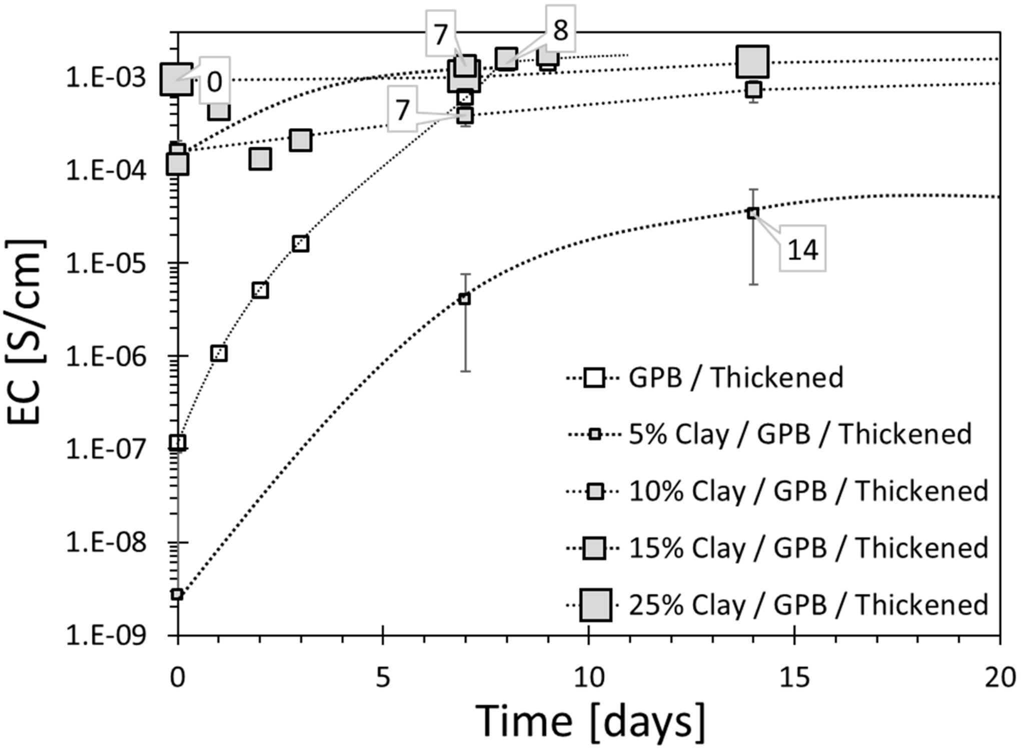

Figure S7. Temporal EC of thickened linseed oil loaded with a fixed GPB concentration of 25 wt% and different concentrations of clay upon exposure to air at 27 ℃. The numbers inserted as labels on the curves are the IAETs of each hybrid system. The dotted lines are drawn as guidance for the eye.
